# Comparative Bioinformatics Characteristic of Bladder Cancer Stage 2 from Stage 4 Expression Profile: A Network-Based Study

**DOI:** 10.22086/gmj.v0i0.1279

**Published:** 2018-12-17

**Authors:** Mona Zamanian Azodi, Mostafa Rezaei Tavirani, Mohammad Rostami-Nejad, Majid Rezaei-Tavirani

**Affiliations:** ^1^Student Research Committee, Proteomics Research Center, Shahid Beheshti University of Medical Sciences, Tehran, Iran; ^2^Proteomics Research Center, Shahid Beheshti University of Medical Sciences, Tehran, Iran; ^3^Gastroenterology and Liver Diseases Research Center, Research Institute for Gastroenterology and Liver Diseases, Shahid Beheshti University of Medical Sciences, Tehran, Iran; ^4^Faculty of Medicine, Iran University of Medical Sciences, Tehran, Iran

**Keywords:** Bladder Neoplasm, Transcriptome, Protein Interaction Maps, Biomarkers

## Abstract

**Background:**

Bladder cancer (BC) has remained as one of the most challenging issues in medicine. The aim of this study was to investigate the differential network analysis of stages 2 and 4 of BC to better understand the molecular pathology of these states.

**Materials and Methods:**

We chose gene expression data of GSE52519 from Gene Expression Omnibus (GEO) database analyzed by the GEO2R online tool. Cytoscape version 3.6.1 and its algorithms are the methods applied for the network construction and investigation of differentially expressed genes (DEG) in these states.

**Result:**

Our result revealed that the analysis DEGs provides useful information about a common molecular feature of stages 2 and 4 of BC.

**Conclusion:**

Consequently, the network finding revealed that more investigation about stage 2 is required to achieve an effective therapeutic protocol to block the transition from stage 2 to stage 4.

## Introduction

Bladder cancer (BC), with the highest rate of death in underdeveloped countries, is the ninth most common type of cancer worldwide [1]. It is also accounted for the second most common type of urological cancer. The risk of BC is higher in men than women [2], with the report of at least 50% recurrence [3]. The available BC detection procedure, though useful, is costly and invasive [4]. In this view, the importance of applying a noninvasive and more sensitive method of detection has led to the application of different molecular investigations, including genomics, transcriptomics, and proteomics profiling. Using these approaches, we aimed to achieve an improved understanding of underlying disease mechanisms and providing a great impact in the clinic [5,6]. There are several molecular investigations about BC that have reported the genes related to the disease. The role of ISG15, GIP2, Cyclin E P53, GSTM1, and several genes in BC is explored and approved [7-10]. Revealing disease-related mechanisms can be effective for developing therapeutic methods [11]. Furthermore, these molecular signatures could also be analyzed by protein interaction mapping via topological examinations. In the network concept, key proteins in a network constitution could be essential to the network integrity and function. For instance, in a disease condition, key proteins that are central to the strength of a network could be dysregulated [12]. As mentioned earlier, one way is to determine the gene expression profile by microarray to find the most key dysregulated genes in the network basis. The differentially expressed genes (DEG) data could be accessible from Gene Expression Omnibus (GEO) database for that specific state of interest, such as different types of cancers by using comparison and network analyzing methods. By doing this, it becomes feasible to provide more validity of identified biomarkers [13]. Following this, the enrichment analysis of the central proteins provides further information. Network analysis, therefore, could present multiple powerful therapeutic targets [14]. In this study, we aimed to examine DEG via protein interaction maps to find better biomarkers for stages 2 and 4 of BC, as bioinformatics can analyze and screen the genes related to various diseases to select the more important ones [15].

## Materials and Methods

To analyze differential and common contributing biomarkers and pathways of BC in stages 2 and 4, the gene expression profiles were examined using bioinformatics. For this purpose, the keyword “Bladder cancer” was searched against the GEO database [16] (www.ncbi.nlm.nih.gov/geo/), and the appropriate study of the mRNA microarray dataset GSE52519 (Genome-wide analysis of gene expression in cancerous and normal human bladder tissues) was downloaded. Moreover, we applied the platform GPL6884, Illumina HumanWG-6 v3.0 expression beadchip, for this study. GEO2R Analyzer was the first bioinformatic study conductor here, which is a Web-based tool in GEO. GEO2R uses the GEO query and limma R packages from the Bioconductor project and provides different statistic information including P value, t test, and fold change for the detected DEGs among samples of interest [17].

The samples were organized into the following 3 groups: healthy (as control), stage 2 of BC, and stage 4 of BC. The dysregulated genes among the cancerous groups were prioritized based on their significant contribution to that specific stage and consequently considered for protein-protein interaction (PPI) network analysis. We used the Cytoscape version 3.6.1. application for the network data visualization and analysis. Network centrality is one of the fundamental concepts for the network [15]. After network data integration via the STRING database plug-in in Cytoscape, essential centrality parameters including degree and betweenness were explored by the use of Genetic Network Analyzer [18,19]. Removal of central nodes can disrupt network communication, which may lead to the abnormal phenotype of a disease [20]. Furthermore, hub-bottlenecks were identified as genes with the highest values of degree and betweenness centrality [21]. To retrieve the functional data on DEGs and hub-bottlenecks in each experimental group of stages 2 and 4, we used GeneMANIA from http://www.genemania.org/plugin/ and Cytoscape platform. This application extracts interaction relations and functional annotation of the corresponding analyzed network [16].

## Result

**Figure 1:**
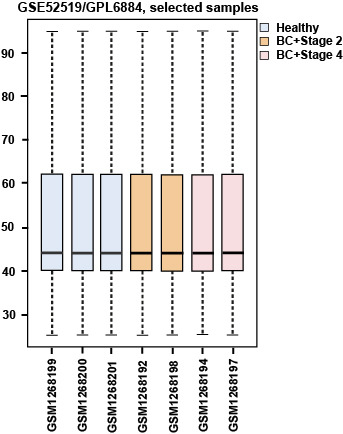
Box plot analysis of gene expression profile for healthy, BC stage 2, and BC stage 4 groups. The healthy sample is indicated in blue, whereas the stages 2 and 4 are shown in orange and pink, respectively. The x-axis denotes the name of sample accession numbers, and the y-axis denotes the range of expression pattern. Samples are median-centered.

To elucidate the distribution of expression values of the 3 compared groups for cross-comparison, we used box plotting. Box plot analysis is available through the GEO2R Online Analyzer (see Figure-1). The finding indicates that the defined groups are comparable.

**Figure 2:**
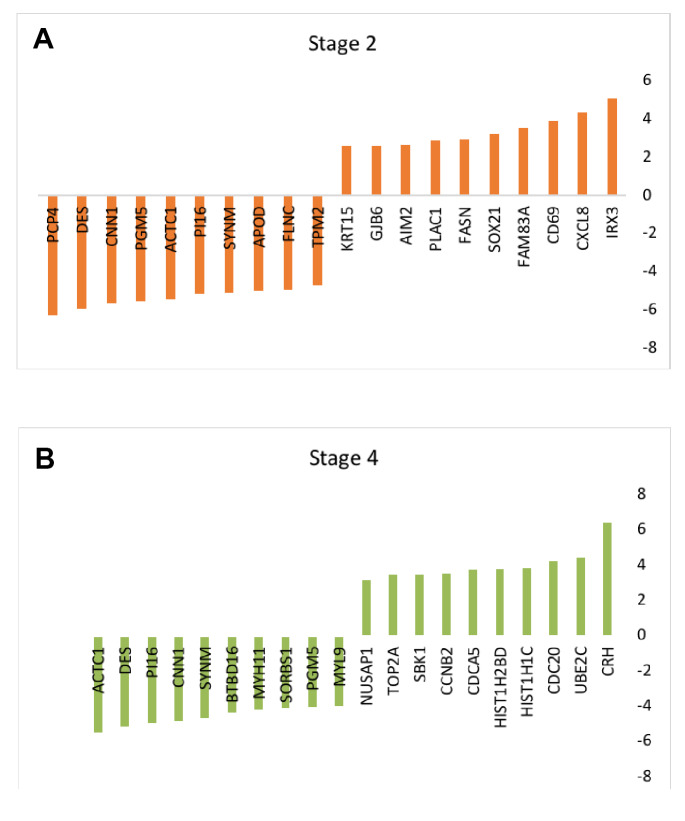
Expression pattern of the top 10 down-regulated and up-regulated genes in stage 2 **(A)** and stage 4 **(B)** of BC. Vertical axis refers to fold change.

Among 250 top genes in terms of differential expression in stages 2 and 4 of BC, 206 and 178 genes, respectively, showed significant differential changes (adjusted P value ≤.05 and FC ≥ 2). The Benjamini-Hochberg procedure (false discovery rate) was used as the default option (the data are not shown) for the adjustment test for P-value. Among these genes, the top 10 up-regulated and down-regulated genes in stages 2 and 4 are listed in Figure-2.

The related networks of BC stages 2 and 4 were constructed. In these networks, the physical interaction was used for centrality analysis. The obtained network of stage 2, including DEGs and 50 added neighbors, consists of 241 nodes and 1771 links. This query has both a linked component and individual ones. To analyze the network, we conducted subnetwork creation and extracted the first component consisting of 183 nodes and 1771 connections. Another network was made of significantly expressed genes in stage 4 of BC compared with control. This network comprised 278 nodes (DEGs plus 50 neighbors) and 4605 links.

**Figure 3:**
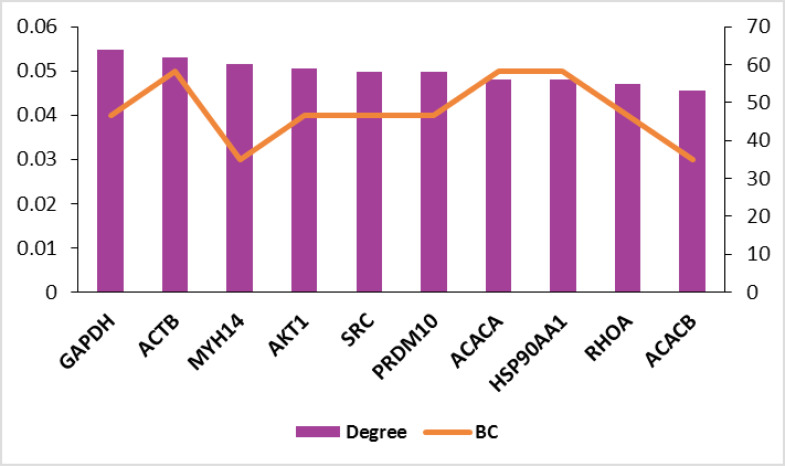
The distribution of degree (right vertical axis) and betweenness centrality (left vertical axis) values of hub-bottlenecks at stage 2 of BC network are shown.

**Figure 4:**
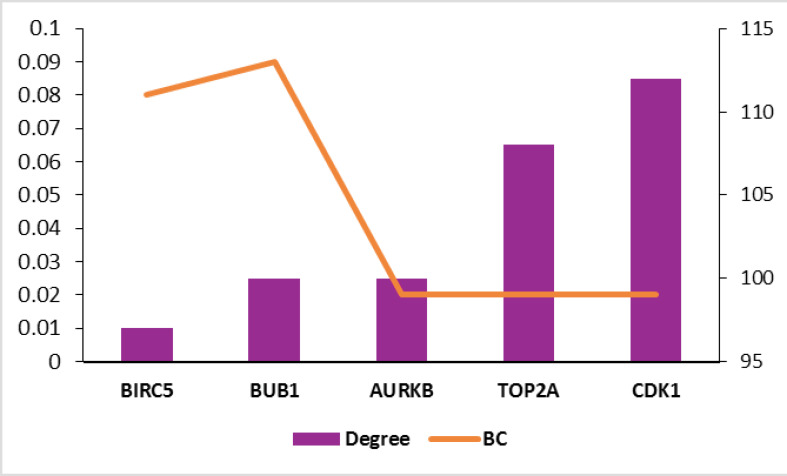
The distribution of degree (right vertical axis) and betweenness centrality (left vertical axis) values of hub-bottlenecks at stage 4 of BC network are shown.

To analyze the whole interacting system, a subnetwork of 190 nodes and 4601 links were obtained as a connected component (the data are not shown). The parameters considered for network analysis are degree and betweenness centrality. The detailed interaction analysis for stages 2 and 4 are shown in Figures-3 and 4, respectively.

**Figure 5:**
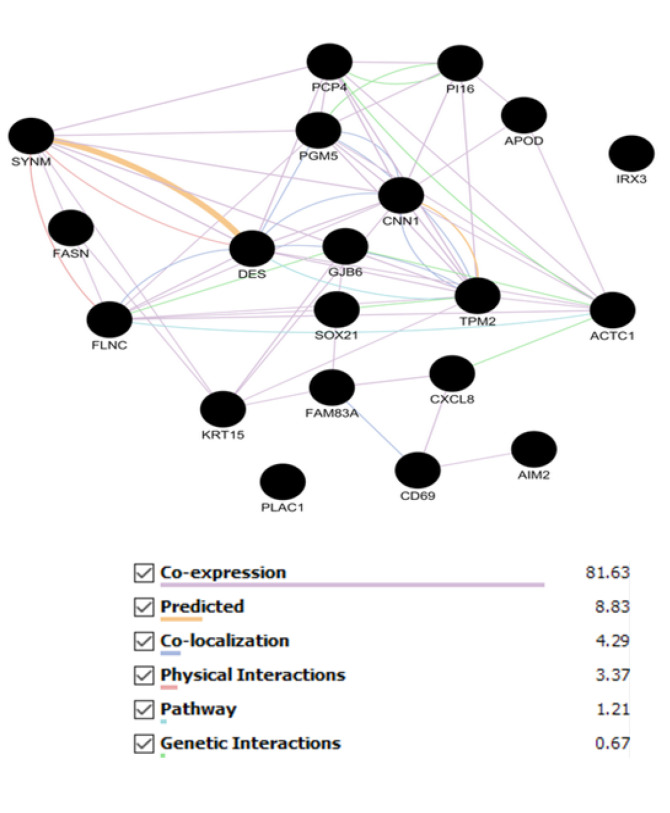
A GeneMANIA network of top differentially expressed genes (DEGs) in bladder cancer (BC) stage 2 consists of 20 nodes and 65 connections. The connections have different relationships between the genes, as shown with different colors. The type and percentage of interactions are mentioned in the figure.

**Figure 6:**
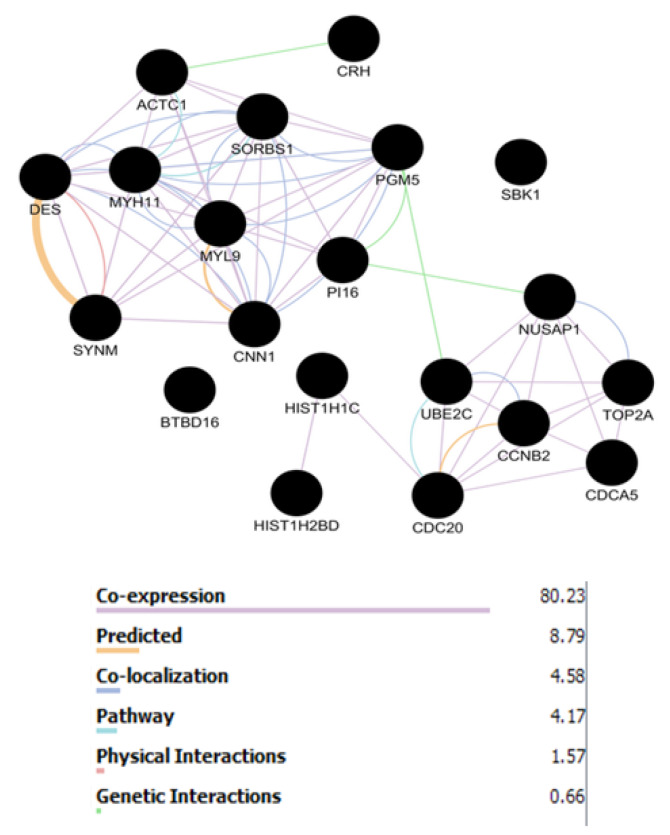
A GeneMANIA network of top differentially expressed genes (DEGs) in bladder cancer (BC) stage 4 consists of 20 nodes and 65 connections. The connections have different relationships between the genes, as shown with different colors. The type and percentage of interactions are mentioned in the figure.

As the mode of interaction can provide useful information, the network of top DEGs of both the stages was constructed based on pathway contribution, coexpression, colocalization, physical interaction, genetic interaction, and predicted functional relations via GeneMANIA (see Figures-5 and 6).

## Discussion

Recent molecular studies have a major impact on disease clarification and are thus helpful for treatment approaches. Prioritizing gene sets related to a specific type of disease in terms of centrality features provides additional information for that disease state [22]. In this study, by studying significantly changed genes regarding their network properties, we prioritized them based on centrality role in the network strength. As indicated in Figure-1, samples are comparable [23] in terms of expression values, and so, the analysis can be done. The comparison shows that there are 6 common genes including PGM5, SYNM, CNN1, PI16, DES, and ACTC1 with differential expression values between stages 2 and 4. Surprisingly, all the common genes are down-regulated. Phosphoglucomutase is a metabolic enzyme that is involved in carbohydrate metabolism [24], and synemin is known as a cell adhesion and cell motility modulator [25]. Association of SYNM with early tumor relapse is investigated and reported [25]. The other 2 common genes Calponin (CNN1) and PI16 are related to the muscular cells [26,27]. The role of desmin in skeletal and cardiac muscle and also ACTC1 in heart function are described in detail [28,29]. It is observed that muscular function is the most common feature of the 2 studied stages. Centrality analysis of the significant DEGs in stages 2 and 4 followed by network construction in Figures-3 and 4 indicated that although none of the central genes in stage 2 are from the query ones, all of the critical genes of stage 4, except CDK1, are among DEGs. The presence or absence of query genes among the central nodes of the PPI network can be related to the information about their properties in the databases [30].

TOP2A, AURKB, BUB1, and BRIC5 are among the dysregulated genes. In addition, AURKB is up-regulated in both stages 2 and 4. It is not included in the selected top 10 changed expression genes, and it is not among the central genes of stage 2.

It is reported that high levels of AURKB are associated with cytogenetic abnormality [31]. Further evaluations of central genes in stage 4 indicated that all the 4 hub-bottlenecks are up-regulated.

These findings show that up-regulation is dominant among key genes of stage 4 and can be considered as a differential point between the stages.

On the other hand, down-regulation is a common aspect between top DEGs of stages 2 and 4. Analyzing top DEGs of stages 2 and 4 as networks in Figures-5 and 6, respectively, indicates that coexpression is a dominant relationship between the genes. The 2 constructed networks are characterized by about 80% expression edges and a range of 1% to 4% physical interactions.

This finding from the GeneMANIA network is in accordance with our finding from gene expression profiling of BC in different stages and further study by PPI network via the STRING database. As it was described in the Methods section, the PPI networks were constructed by additional 50 relevant genes. The poor interactions between the query genes required additional nodes to constitute the interactive units for stages 2 and 4 of BC. However, the pathway relationships between the 2 stages are inconsiderable in comparison with expression links; stage 2 is not as good as stage 4. Relative similarity between top DEGs and the central nodes is reported in other documents [32].

This finding refers to unmatched roles of the elements of stage 2 network relative to the nodes of stage 4 interactome. The main findings of this research can be summarized as follows:

1. The muscular system is the most involved tissue in stages 2 and 4 of BC.
2. There are large amounts of information about the involved elements of stage 4 relative to stage 2 of BC in the databases.
3. Up-regulation of the critical genes is the key feature of stage 4, whereas down-regulation of the top DEGs is the common process for the 2 studied stages.
4. Coexpression is the dominating relationship between DEGs in both the stages.

Therefore, it is possible to introduce a differential method for the 2 stages, which may be useful in early-stage diagnosis of the disease. Although magnetic resonance imaging and pathology are used frequently to analyze the stage of different cancers [33], the molecular finding can improve the staging methods effectively and economically. On the basis of our finding, stages 2 and 4 of BC can be differentiated, but more investigation, such as research in the field, is needed.

## Conclusion

It can be concluded that based on DEG, there are significant common molecular maps and patterns between stages 2 and 4 of BC. PPI network analysis revealed the top DEGs as central genes in stage 4, but it showed the difference between DEGs and central genes of stage 2 BC. Therefore, more investigation about the molecular mechanism of stage 2 is required, which could be useful in the prevention of transition from stage 2 to 4 in BC.

## Conflict of Interest

There is no conflict of interest
